# Organizing the Chaos: Novel Insights into the Regulation of Z-3-Hexenal Production in Damaged Maize Leaves

**DOI:** 10.3390/plants13192772

**Published:** 2024-10-03

**Authors:** Samantha Selman, Marie Engelberth, Jurgen Engelberth

**Affiliations:** 1 Department of Plant Pathology, Texas A&M University, College Station, TX 77843, USA; samantha.selman@agnet.tamu.edu; 2 Department of Integrative Biology, University of Texas at San Antonio, San Antonio, TX 78249, USA; marie.engelberth@utsa.edu

**Keywords:** green leaf volatiles, calcium, hydrophobic cluster, biosynthesis, fatty acids, hexenal

## Abstract

Green leaf volatiles (GLVs) are important signaling compounds that help to regulate plant defenses against pests and pathogens. Made through the hydroperoxide lyase (HPL) pathway, they are rapidly produced upon damage and can signal to other parts of the same plant or even plants nearby, where they can induce rapid defense responses directly or prime them against impending danger. In this primed state, plants can respond faster and/or stronger should pests or pathogens attack. However, while all proteins and genes involved in the biosynthesis of GLVs have been identified, little is still known about how the first two steps in the pathway, e.g., oxygenation by a lipoxygenase (LOX) and subsequent cleavage by HPL, are facilitated within the damaged tissue, resulting in the production of Z-3-hexenal (Z3al) as the first committed product of the pathway. Here, we provide evidence that several factors might be involved in the production of Z3al, including pH, Ca^2+^, and an environment that is highly hydrophobic. We present a model in which the extraordinary circumstances that are present at the site of Z3al production are considered, and shine new light on potential regulatory mechanisms.

## 1. Introduction

Green leaf volatiles (GLVs) are a ubiquitous group of plant compounds that have been known for more than 100 years [1]. However, while originally considered to be metabolic shunts, they have come to our attention as potent inducers of plant protective measures, covering both microbes and herbivores [2,3]. In more recent years they have further been found to also protect against a range of abiotic stresses including drought, cold, high light, and heat [4]. Common to all these threats is that they can cause significant damage to the plant, which is also the main contributor to the production and release of GLVs.

The biosynthesis of GLVs starts with a lipoxygenase (LOX) that inserts molecular oxygen into a fatty acid, in the case of GLVs in position 13 [5,6]. While this can be a free fatty acid, it has been demonstrated that lipid-bound fatty acid can also serve as a substrate [7]. The hydroperoxyl fatty acid, which is usually linolenic or linoleic acid, is then further processed by a hydroperoxide lyase (HPL) and cleaved into a 6-carbon unit and a 12-carbon unit. While the 12-carbon unit can be transformed into traumatin (12-oxo-trans-10-dodecenoic acid) [8], the 6-carbon unit, which is either Z-3-hexenal (Z3al, from linolenic acid) or hexanal (Hal, from linoleic acid), represents the first GLV compound in the pathway. Z3al in particular can be produced in large quantities from damaged plant tissue, while Hal is usually a minor compound [9]. Both can be further processed into the corresponding alcohols by an alcohol dehydrogenase and eventually into esters by an acetyl transferase [5,6]. Some plants also have an isomerase, which converts Z3al into E-2-hexenal (E2al) [10,11]. E2al can also be reduced to the corresponding alcohol and esterified [5,6]. The production of the aldehydes including the isomerase reaction occurs in damaged tissues, while the biosynthesis of the alcohols and esters requires intact cells [12].

However, GLVs are also released from undamaged plants under certain biotic and abiotic stresses. For example, it was shown that drought and high temperatures can cause the release of significant amounts of these compounds from intact plants, which are therefore mainly found in the form of the corresponding alcohols and esters [13]. Others have observed a burst of GLVs from intact plants right after the onset of darkness, again mainly as alcohols and esters [14,15]. Also, plants experiencing either insect herbivory or pathogen infections often release GLVs from distal or systemic undamaged parts, although usually with some delay [16].

To date, no receptors for GLVs have been identified. However, progress has been made in the characterization of signaling events activated by these compounds. Several signaling pathways related to GLV activities have already been elucidated, including rapid changes in membrane potential and altered cytosolic Ca^2+^ levels [17,18]. An array of transcription factors that are induced by exposure to GLVs have been identified [19,20,21]. It is, however, unclear if these are GLV-specific and what the downstream targets are. Most recently, in tomato (*Solanum lycopersicum*), a MAP kinase pathway was characterized that responded to GLV treatment [22]. Interestingly, the same MAP kinase pathway is also recruited upon pathogen infection. Z-3-hexenol (Z3ol) can also be conjugated to other cellular molecules, in particular sugars [2]. Several different forms of this conjugation have been identified and were also shown to have anti-herbivore properties [23]. Also, GLV-glycosides can easily become hydrolyzed to release Z3ol again. Treatment with Z-3-hexenyl acetate (Z3ac) also results in the formation of these glycosides, implying that Z3ac first has to be cleaved into Z3ol before conjugation. This further suggests that Z3ol may also be the active compound among the GLVs, which was confirmed by [24] in showing that mutations in the hydrolyzing enzymes reduced the activity of Z3ac significantly. In contrast, E2al has been found to become conjugated to glutathione as a detoxification mechanism, rather than being a storage product [25].

Since physical damage to green plant tissues can cause the production and release of these compounds almost instantly, they are ideal volatile messengers to report the damage to distant yet undamaged parts of the same plant, as well as to other plants nearby. There, they may alert these to the possibility of impending damage [2]. Adding to this is the ability of many plants to produce large quantities of GLVs, which may reach several µg per gram of fresh weight [9]. GLVs can further be produced in different isomers. While some plants mainly produce the *Z*-3 isomers, others predominantly produce the E-2 isomers [9]. Even within one plant species the quality and quantity of produced GLVs can vary significantly depending on environmental factors and developmental stages [26]. This diversity of GLV production and release contributes to the complexity of their biology and implies that ecophysiological factors may have played an important role in the evolutionary shaping of GLV activities [9].

Common to the events that cause the release of GLVs is the association with tissue damage. Therefore, the starting point for the biosynthesis of GLVs lies within the damaged tissue itself, with Z3al being the first product of the pathway. This implies a mechanism that allows LOX (for maize LOX10 [27]) and HPL (for maize [28]) enzymes in particular to become active in damaged cells, either by eliminating spatial separation normally found in intact cells, or by changing the general reaction conditions. Also, within the damaged tissue both enzymes need access to their substrates, which can either be found in the form of vesicles, individual lipids, or free fatty acids [2,5,6,7]. However, little is known about these processes. We therefore started an investigation with the aim to identify factors that may help to facilitate the rapid production of Z3al as the first distinct compound of the pathway. Multiple parameters, including pH, specific ions, pharmacological inhibitors, as well as the presence or absence of polar and unpolar components, were investigated. Furthermore, we performed a proteomics analysis of thylakoid membranes isolated from maize chloroplasts and stroma to determine the major location of LOX10 and HPL. Together, a picture emerges that helps to explain how plants may regulate GLV production in damaged tissues without the constraints of strict spatial separation.

## 2. Results and Discussion

### 2.1. Identification of LOX10 and HPL in Thylakoid Membranes of Maize Chloroplasts

LOX10 in maize has been thoroughly characterized [27,29], while HPL has been much more elusive. Only recently did Yactayo-Chang et al. identify and characterize the *HPL* gene in maize [28]. However, while both LOX10 and HPL proteins were predicted to be in chloroplasts, the precise location within those was still unknown. To find out more about the subcellular localization of LOX10 and HPL in maize leaf cells, we isolated chloroplasts from leaf tissue first, followed by an enrichment of thylakoid membranes. The enriched thylakoid samples were then analyzed in the Mass Spectrometry and Proteomics Core at UTSA (https://research.utsa.edu/cores/mspc/ (accessed on 7 May 2021)). As expected, while almost all proteins identified in the enriched thylakoid sample were involved in photosynthesis, only a few other proteins with no direct involvement in photosynthesis were identified, among them LOX10 and HPL with LOX10 showing a slightly higher abundance (emPAI 0.45) as HPL (emPAI 0.4). We also identified a lipase in the thylakoid fraction, although at a much lower abundance (emPAI 0.09) (Table 1).

We did not identify any other proteins involved in oxylipin metabolism in the thylakoids, suggesting that those are mostly localized in either the stroma or the outer membrane areas. The data strongly support the notion that LOX10 and HPL are co-localized in the thylakoid membrane system of maize leaf chloroplasts. This is in accordance with previous results on the localization of these enzymes. For example, while HPL in potato (*Solanum tuberosum*) was also found in thylakoid membranes [30], other HPLs (e.g., spinach (*Spinacia oleracea*), tomato, and *Arabidopsis thaliana*) have been found in the envelope membrane system [31,32,33]. Likewise, LOX enzymes can be localized in different compartments at the cell and organelle level as well [34,35,36]. Nonetheless, in maize both enzymes appear to be co-localized in thylakoids, which might be the key to their rapid activation upon damage or other stressful events [2]. The identified lipase appears to belong to the phospholipase A type family with similarities to the Arabidopsis DAD1 lipase [37] and might be involved in the release of traumatin from these membranes, as reported in [7], since no other oxylipin-related enzymes were identified. However, although LOX10 and HPL appear to be co-localized within the thylakoid membrane system in maize chloroplasts, this does not explain how they become activated upon damage.

### 2.2. Z3al Production Strongly Depends on the pH of the Environment

**Figure 1:**
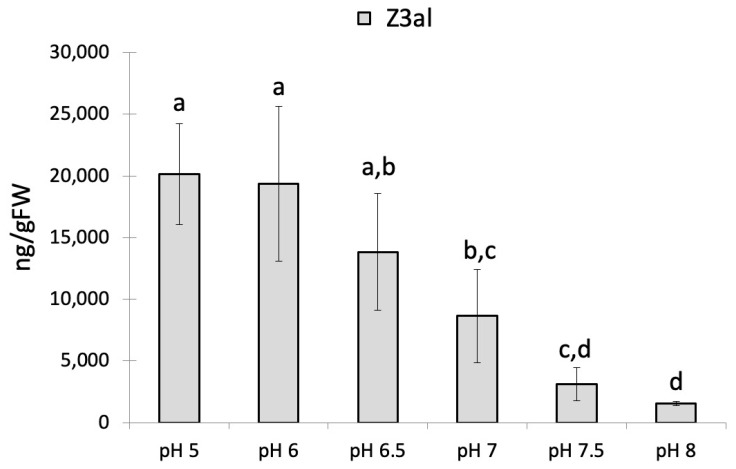
The effect of pH on the production of Z-3-hexenal (Z3al) in a cell extract. Plant material was taken from the same leaves of different maize plants of the same age (12 days) and developmental stage (late V2, third leaf was used). Different letters above each bar indicate statistical differences determined by ANOVA analysis followed by Tukey tests where appropriate (*p* < 0.05). *N* = 4, error bars represent standard deviation.

We first determined the dependency of Z3al production on pH in damaged maize leaf tissues. We used a potassium phosphate (KPi) buffer system since it covers most of the expected pH range. We found maximum production activity for the combined LOX10/HPL system at acidic pH levels of 5 (20,142 ± 4095 ng/gFW) and 6 (19,347 ± 6272 ng/gFW). Beginning at pH 6.5, activity started to decline rapidly, and at pH 8, less than 10% (1531 ± 181 ng/gFW) of the initial activity was detected (Figure 1).

This is in accordance with pH measurements of damaged tissue from corn leaves, which were found to be between pH 5 and pH 6. Therefore, the optimum pH to produce Z3al coincides well with what is found within the special context of damage. As such, pH appears to be an important factor in the activation of the LOX10/HPL system. However, the optimum pH for LOX10 was reported to be around pH 8, although a significant activity was still measured at lower pH, indicating a certain biosynthetic plasticity regarding this parameter [27,29].

HPLs often appear to have a pH optimum between 5 and 6 [30,38,39,40]. While this does suggest that HPL is the key enzyme in this enzymatic complex upon tissue damage due to rapid changes in pH, a prominent role for LOX10 despite the different pH optimums cannot be ruled out. LOX10 activity may therefore also depend on other factors, e.g., substrate availability, substrate affinity, the effectiveness of the reactive center, and others. It is therefore necessary for future experiments to further investigate the individual enzymatic parameters for both enzymes in more detail and study their potential for direct interactions as well.

The observed acidic optimum for the production of Z3al also sheds further light on findings that describe how insect herbivores suppress the production of Z3al while feeding on plants [41,42,43,44,45]. Among the mechanisms described were enzymes that eliminated the substrate for HPL, a yet-to-be-identified molecule that appears to bind Z3al, and an isomerase that rapidly transforms Z3al into E2al. All insect herbivores studied to date have at least one of these mechanisms abundant in their saliva and can thus apply them immediately to the freshly eaten plant material to suppress Z3al production [45]. However, in most cases, this saliva was also found to be very basic (pH around 9 and higher), and this alone would already suppress the activity of the LOX10/HPL system, thereby reducing the amount of Z3al produced in the freshly ingested tissue significantly.

The effects of pH on GLV biosynthesis may also explain why at least some plants, when placed in the dark, release a burst of GLVs [14,15]. Darkness may quickly change the pH gradient across the thylakoid membrane due to the inactivation of PSII and may therefore result in a more acidic region on the stroma side due to the halted influx of protons into the thylakoid lumen and the simultaneous outflow of H^+^ through the ATP-synthase complex. This spatial and transitional acidification then activates the LOX10/HPL complex and consequently results in a sharp burst of GLV emissions. However, further evidence is needed to prove this hypothesis. Nonetheless, our results clearly demonstrate that pH is an important factor in the activation of the LOX10/HPL system. Therefore, all further experiments were performed at pH 6.

### 2.3. Phenidone Is an Effective Inhibitor of Z3al Production

**Figure 2:**
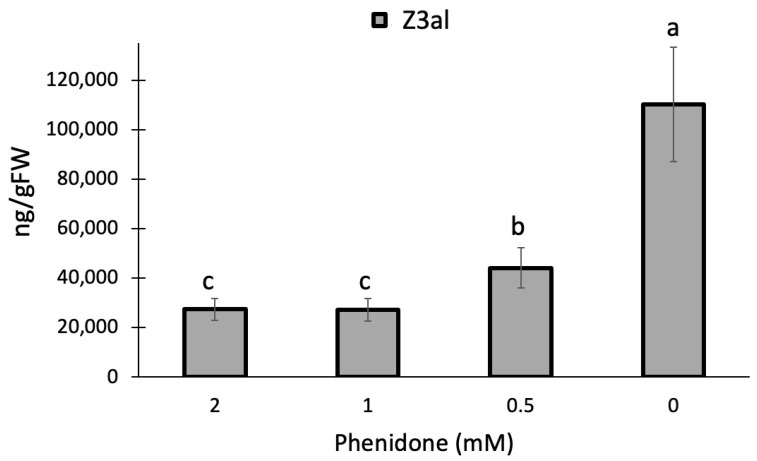
Phenidone reduces the production of Z-3-hexenal (Z3al) in a cell extract. Plant material was taken from the same leaves (3rd) of different maize plants of the same age (16 days) and developmental stage (V3). Phenidone was pre-dissolved in ethanol and then added to the final concentration indicated in the graph. Control samples received an identical concentration of ethanol only. Different letters above each bar indicate statistical differences determined by ANOVA analysis followed by Tukey tests where appropriate (*p* < 0.05). *N* = 4, error bars represent standard deviation.

While HPL in the HPL/LOX10 system appears to fit better into the pH response, we used a pharmacological approach to further assess the role of LOX10 in the process of producing Z3al in damaged tissue. Phenidone is an effective inhibitor of lipoxygenase activity and has been shown to significantly reduce jasmonic acid biosynthesis in maize [46] and other plants through this mechanism [47,48,49]. Since it inhibits all lipoxygenases, including LOX10, we expected it to negatively affect the production of Z3al when added to our assay. We used concentrations ranging from 0.5 mM to 2 mM as well as a buffer control for testing. We found a significant effect of phenidone on Z3al production in our in vitro assay. A concentration of 0.5 mM phenidone in the buffer already reduced the activity of the system by more than 50% (buffer only 110,260 ± 23,175 ng/gFW, 0.5 mM phenidone 44,064 ± 8095 ng/gFW) (Figure 2). However, higher concentrations (1 mM and 2 mM) leveled out at 75% inhibition.

The significant reduction in Z3al production by phenidone suggests a de novo synthesis of Z3al upon damage with LOX10 being a rate-limiting factor despite its pH optimum in the more basic range. However, some oxygenated fatty acids may already be abundant in the damaged leaf, as indicated by the 25% rest activity in phenidone-treated samples. Since maize contains 13 lipoxygenases, 6 of which are 13-lipoxygenases [50], some background activity cannot be excluded.

### 2.4. Effect of Ions on Z-3-Hexenal Production in Maize

**Figure 3:**
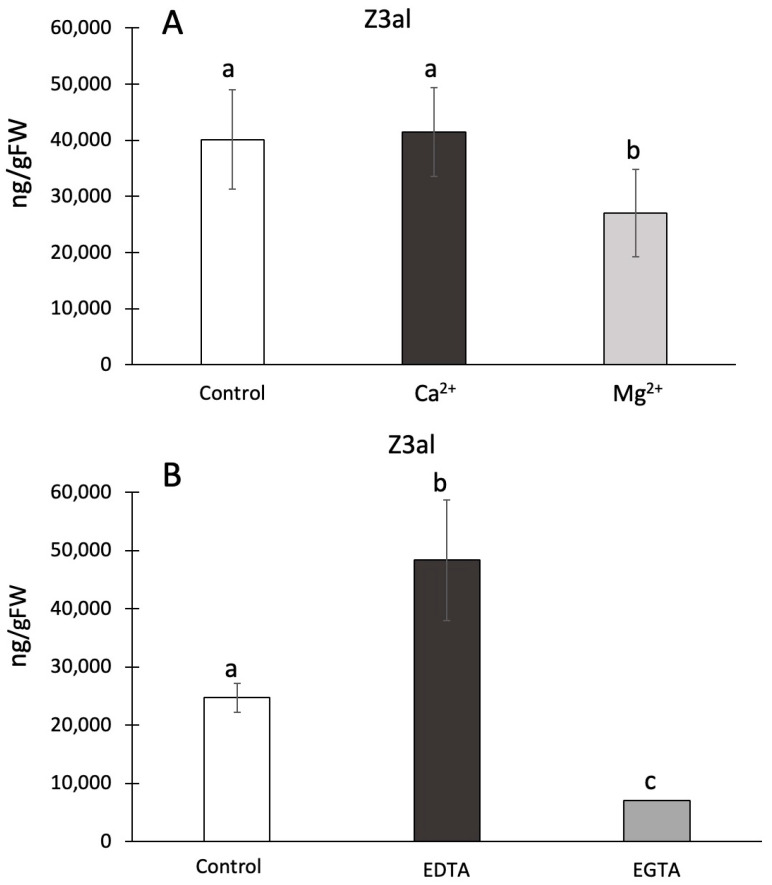
Effects of divalent ions and chelators on Z-3-Hexenal (Z3al) production. Plant material was taken from the same leaves (3rd) of different maize plants of the same age (14 days) and developmental stage (V3). (**A**), divalent ions were added to a final concentration of 10 mM in phosphate buffer at pH 6. (**B**), chelators were dissolved at a basic pH and then titrated down to pH 6. Chelators were added to the sample to a final concentration of 10 mM. Different letters above each bar indicate statistical differences determined by ANOVA analysis followed by Tukey tests where appropriate (*p* < 0.05). *N* = 4, error bars represent standard deviation.

Ca^2+^ has been previously implied to play a role in the activation of the initial steps of GLV biosynthesis [51] by binding to the lipoxygenase and facilitating its association with membranes [52]. To test the hypothesis of Ca^2+^ being relevant for this process in maize we used several different assays, including the addition of Ca^2+^ as well as its complexing through the addition of EGTA. We also tested for the effects of other ions, including Mg^2+^ and Na^+^. Divalent ions were added to the reaction mix at different concentrations, but ultimately 10 mM was used in our in vitro assays. We found that Ca^2+^ had no effect on the capacity to produce Z3al when compared to control assays (41,437 ± 7888 ng/gFW vs 40,142 ± 8892 ng/gFW) (Figure 3A). While this was surprising, it is also worth noting that disrupted plant tissue, as it is used in our assays, may contain significant amounts of free Ca^2+^, which is usually strictly compartmentalized. However, once released by damage, the overall concentration could be sufficient to activate LOX10. In contrast, Mg^2+^ caused a 40% reduction in Z3al production (26,985 ± 7795 ng/gFW) (Figure 3A). Using higher concentrations of Mg^2+^ in the assays did not change this ratio.

To further study the effects of divalent ions on GLV production in damaged maize leaf tissue, we used ethylene glycol-bis(β-aminoethyl ether)-N,N,N′,N′-tetraacetic acid (EGTA) and ethylenediaminetetraacetic acid (EDTA) to remove these ions from the reaction mix [52]. EGTA has a higher preference for Ca^2+^ ions, while EDTA prefers Mg^2+^ [53,54]. We found that by adding 100 mM of EDTA, the capacity to produce Z3al was increased by almost 100%, indirectly confirming the inhibitory activity of Mg^2+^ on the reaction (H_2_O control 24,690 ± 2494 ng/gFW; EDTA 48,310 ± 10,410 ng/gFW) (Figure 3B). In contrast, adding the same concentration of EGTA caused a significant reduction in Z3al production by more than 70% (7005 ± 893 ng/gFW). In contrast to our finding that added Ca^2+^ had no effect on the biosynthesis of Z3al, the removal of Ca^2+^ did have an inhibitory effect. As stated before, it might still be the case that endogenous Ca^2+^ is sufficient to saturate the activating process immediately upon damage, thereby causing the association of LOX10 and/or HPL to membranes. Any further added Ca^2+^ will no longer provide an additional activation of the system. However, when we analyzed LOX10 and HPL for potential ion binding sites at IonCom (https://zhanggroup.org/IonCom/ (accessed on 14 April 2024)) [55], no potential binding sites for Ca^2+^ or Mg^2+^ were detected on either protein. We can therefore only assume that Ca^2+^ might be more involved in the association of membranes in the damaged tissue, which may lead to an increased supply of membrane lipids to the Z3al-producing complex. Ca^2+^ is well known to be a regulator of membrane assembly and it might be that this is the main reason why Ca^2+^ is one of the factors that regulate or even stimulate Z3al production [51].

**Figure 4:**
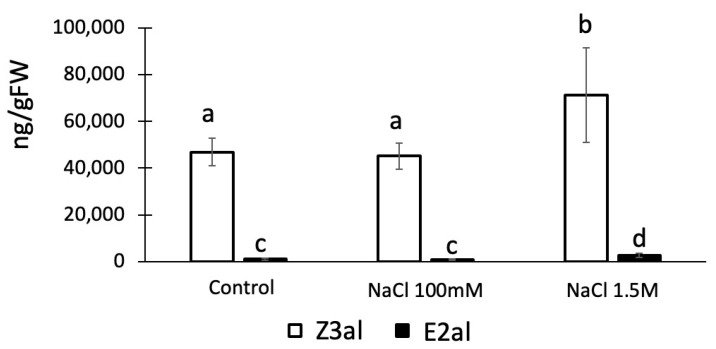
Effects of low and high sodium chloride (NaCl) concentrations on the production of Z-3-hexenal (Z3al) and E-2-hexenal (E2al) in a cell extract. Plant material was taken from the same leaves of different maize plants of the same age (16 days) and developmental stage (V3, third leaf was used). Different letters above each bar indicate statistical differences determined by ANOVA analysis followed by Tukey tests where appropriate (*p* < 0.05). *N* = 4, error bars represent standard deviation.

We further tested the effects of Na^+^ as a monovalent ion on the production of Z3al. We found that Na^+^ at concentrations similar to those used for Ca^2+^ and Mg^2+^ had no effect on the activity of the Z3al-producing system (48,689 ± 8054 ng/gFW in control vs 45,107 ± 5660 ng/gFW in Na^+^ treated samples) (Figure 4). However, increasing the concentration of Na^+^ to 1.5 M in the assays significantly increased Z3al production from 48,689 ± 8054 ng/FW to 71,284 ± 18,239 ng/gFW, an increase of 46%. We do not attribute this increase to a specific effect of the Na^+^ ion, but rather hypothesize that a concentration this high may force lipids, fatty acids, and the LOX10/HPL complex closer together resulting in a hydrophobic complex or cluster that allows for increased activity of the system.

### 2.5. Effects of Fatty Acids and Detergent on GLV Production

**Figure 5:**
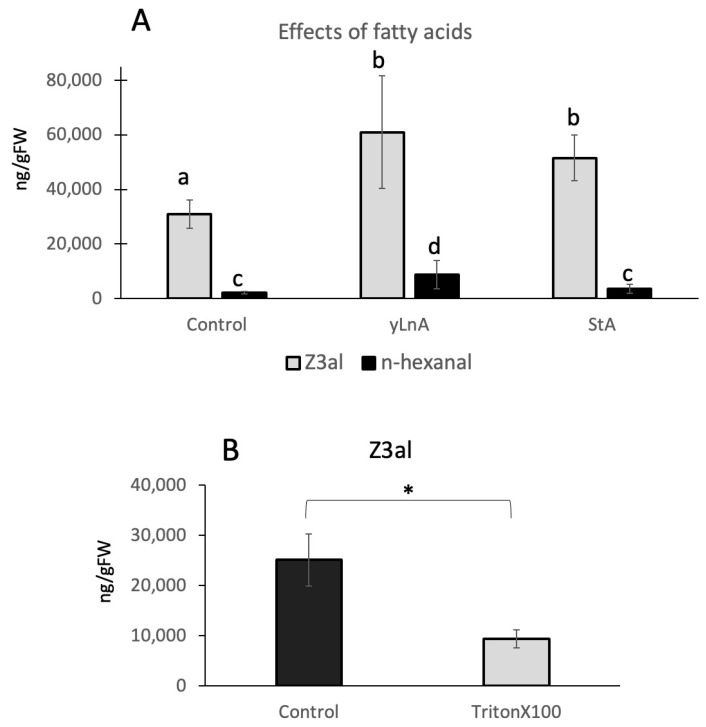
Effects of fatty acids and a detergent (TritonX100) on the production of Z-3-hexenal (Z3al) in a cell extract. (**A**), fatty acids (gamma-linolenic acid (yLnA) and stearic acid (StA)) were added to a final concentration of 1 μg/μL. Also displayed is n-hexanal. (**B**), Triton X100 was added at 0.1% to the samples before tissue disruption. (**A**), different letters above each bar indicate statistical differences determined by ANOVA analysis followed by Tukey tests where appropriate (*p* < 0.05). (**B**), a *t*-test was used for pairwise comparison and significant differences are indicated by *. *N* = 4, error bars represent standard deviation.

To further test the hypothesis that hydrophobic cluster formation around the LOX10/HPL complex is an important part of the biosynthetic effectiveness of the system, we added free fatty acids to the reaction mix. Since fatty acids with a double bond in position 13 may also serve as substrates, we selected both, γ-linolenic acid and stearic acid, which is an 18:0 fatty acid, in our assays. Both fatty acids were used at a final concentration of 1 μg/μL and were added just before the plant tissue was damaged and subsequently analyzed. We found that both fatty acids significantly increased the amount of Z3al by 96% for γ-linolenic acid (60,927 ± 20,626 ng/gFW) and 66% for stearic acid (51,579 ± 8416 ng/gFw) when compared to the control (31,041 ± 5161 ng/gFW) (Figure 5A). Since γ-linolenic would also yield hexanal in the reaction, we monitored this compound too. While levels were significantly higher in γ-linolenic samples (8847 ± 5156 ng/gFW compared to control 2133 ± 472 ng/gFW), only minor increases were found for stearic acid (3669 ± 1669) (Figure 5A), which can however not be attributed to stearic acid itself since it cannot serve as a substrate for the reaction. It can therefore be concluded that although γ-linolenic increased hexanal production, its addition also stimulated Z3al production directly, as did stearic acid, which suggests that the presence of these fatty acids has a more general effect on the reaction rather than just serving as substrates.

The results also provide support to our hypothesis that the creation of a hydrophobic environment together with a sufficient supply of substrate might be essential for the amount of GLVs that can be produced. While thylakoid membranes are very rich in lipids and thus fatty acids, this may ensure that sufficient amounts of those are made available for the reaction by potentially clustering together. To further test this hypothesis, we used a detergent (Triton X-100) in a similar assay. Adding a detergent should cause the dissociation of such a hydrophobic clustering and thus reduce Z3al production significantly. As expected, when added at a final concentration of 0.1%, Triton X-100 reduced the activity of the Z3al-producing system significantly by more than 60% (25,081 ± 5176 ng/gFW control, 9366 ± 1739 g/gFW Triton X100) (Figure 5B). This finding therefore backs our hypothesis that hydrophobic clusters are being formed in damaged tissue, which ensures that a sufficient amount of substrates are available for the reactions leading to Z3al production.

## 3. Summary

The co-localization of LOX10 and HPL in thylakoids was confirmed by a proteomics approach with isolated thylakoid membranes. Aside from a yet-to-be-characterized lipase, no other proteins not involved in photosynthesis were detected.

Damaged maize leaf tissues provide an optimized environment for the production of GLVs through their acidic pH, which allows them to serve as a volatile signal for other parts of the plant or even plants nearby. This can provide far-reaching protection for those plants against damaging stresses, including herbivory and pathogen infections [2], and a great variety of abiotic stresses like cold, drought, and light [4]. For insect herbivores, the strong dependency on Z3al-biosynthesis in damaged maize leaves in an acidic environment provides another path toward blocking the production of GLVs. Since most insect herbivores appear to have a very basic pH in their spit and gut system, this may add to their arsenal of countermeasures aimed toward a reduction in GLV biosynthesis, thus eliminating a signal that eavesdropping plants nearby could use to prime their defenses.

While Ca^2+^ clearly affects the production of Z3al, we have no further evidence for potential Ca^2+^-binding sites on either protein. This makes it unlikely for Ca^2^ to act as a regulator for the association of LOX10 or HPL to membranes or for the activation of any one of those enzymes. However, Ca^2+^ has been found to be involved in the regulation of membrane assembly [56], and we may therefore speculate that Ca^2+^ may instead assist in the assembly of hydrophobic structures or complexes that allow LOX10 and HPL to have access to more substrates

**Figure 6:**
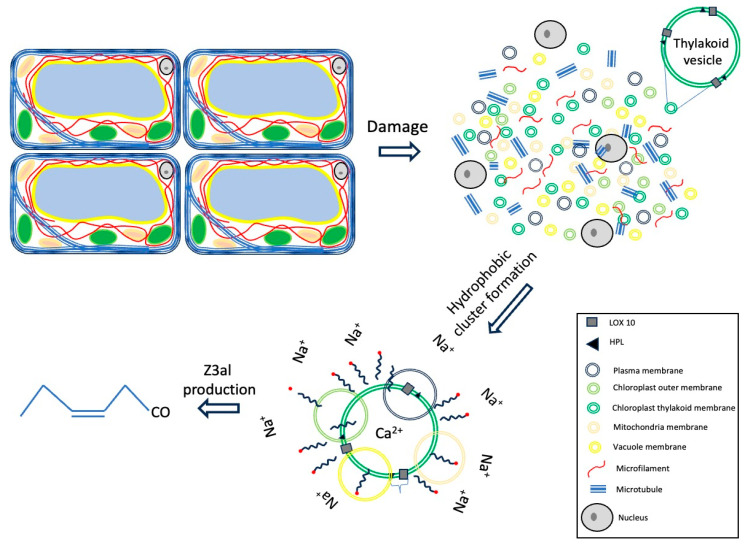
Effects of Ca^2+^, fatty acids, and high salt concentrations on the production of Z-3-hexenal (Z3al). Rings of different colors indicate membranes from different sources which can still contribute to the production of Z-3-hexenal in this model.

Adding free fatty acids to the reaction stimulates the biosynthesis of Z3al in damaged plant tissue. However, while some fatty acids like γ-LnA may also serve as substrates, the results with stearic acid clearly demonstrate that the abundance, rather than substrate availability is a factor that determines the effectiveness of the Z3al-producing system. This is further supported by the effect of Triton X100, which as a detergent would disrupt these hydrophobic complexes. A summary of our findings is shown in Figure 6.

While these clusters may primarily be comprised of thylakoid membranes, other membranes within the damaged plant tissue may also contribute to the overall production of Z3al. However, since Z3al is the most prominent aldehyde compound produced in damaged maize leaf tissue, a certain preference for α-linolenic acid or related omega 3 fatty acids seems to be highly probable. While high salt concentrations appear to further activate the system and support our hypothesis of hydrophobic cluster formation, the required concentrations of these and related ions are rather unlikely to form within damaged leaf tissues.

To summarize, we have separated factors that stimulate Z3al biosynthesis. Based on our findings, we hypothesize that a combination of pH, Ca^2+^-abundance, and hydrophobic cluster formation are important factors that aid in the massive production of Z3al in damaged leaf tissue in maize. These regulatory principles may help to partially organize the otherwise chaotic mixture of cellular components as it occurs upon tissue damage in plants.

## 4. Materials and Methods

### 4.1. Chemicals

(E)-2-hexen-1-al (E2al), was provided by Bedoukian (Bedoukian Research, Danbury, CT, USA). All other chemicals (e.g., (*Z*)-3-hexen-1-al (*Z*3al), n-Hexanal (nHal), nonyl acetate, Ethylene glycol tetraacetic acid (EGTA), Ethylenediaminetetraacetic acid (EDTA), Percoll, Triton X100, and sorbitol) were purchased from Sigma-Aldrich (St. Louis, MO, USA). All solvents used were analytical grade.

### 4.2. Plant Material

Maize (*Zea mays*, var. Kandy King, J.W. Jung Seed Co., Randolf, WI, USA) plants were grown in Sungro Horticulture Professional Growing Mix (Sun Gro Horticulture Canada Ltd., Seba Beach, AB, Canada) in a growth chamber under a 12 h photoperiod at 26 °C with 60% relative humidity. Light intensity was set to ca. 150 μmol m^2^ s^−1^. Maize plants used for the analysis of aldehyde GLV production capacity were between 2 and 3 weeks old and in the late V2 to V3 stage. We used segments from the middle of the leaves for all experiments.

### 4.3. Experimental Setups

#### Chloroplast Isolation and Thylakoid Enrichment

In the first step, intact chloroplasts were isolated by following a method of Bhattacharya et al. [57] with minor modifications. During the preparation, all materials were kept on ice. We first harvested 25 g of leaf material from 2-week-old maize plants, added 95 mL of 1x grinding buffer (GB buffer, 2x-GB buffer 100 mM Hepes-KOH, pH 7.3, 660 mM sorbitol, 2 mM MgCl2, 4 mM EDTA, and 0.2% BSA (*w*/*v*)), and homogenized the mixture in a blender with sharpened blades. After homogenization, the mixture was filtered through several layers of cheesecloth in a funnel. The crude chloroplast extract was then centrifuged at 3000× *g* in a swinging bucket rotor for 5 min at 4 °C. The pellet was gently resuspended with 1 mL of 1x GB buffer and kept on ice. The Percoll gradient consisted of an 80% Percoll layer at the bottom with a 40% Percoll layer on top. Percoll was mixed with 1x GB buffer to the final concentration. The chloroplast pellet was then carefully layered on top of the Percoll gradient and centrifuged at 10,000× *g* in a swinging bucket rotor for 10 min at 4 °C with brakes off. The resulting band between the 80% and 40% Percoll layer was then carefully taken out, diluted in resuspension buffer (RB, 50 mM HEPES-KOH pH 8, 330 mM sorbitol) at a 1:4 ratio, and centrifuged at 4500× *g* in a swinging bucket rotor for 5 min at 4 °C. The supernatant was discarded, and the pellet was resuspended in RB buffer and centrifuged again. The final pellet was resuspended again in 1x GB buffer and used for thylakoid isolation.

Thylakoid membranes were prepared by hypotonic lysis of the intact chloroplasts following the protocol of Bouchnak et al. [58] with modifications. Chloroplasts were centrifuged as before (1800× *g*, 5 min, 4 °C) and then mixed with 3 mL of the lysis buffer (MOPS (10 mM, pH 7.8), MgCl_2_ (4 mM), PMSF (1 mM)) and incubated on ice for 15 min. Samples were vortexed repeatedly during the incubation. After lysis, 3 mL of the incubation solution was placed on a sucrose gradient. The gradient consists of 3 layers, 3 mL of 0.93 M at the bottom, then 2.5 mL of 0.6 M, and 2 mL of 0.3 M sucrose. Sucrose was dissolved in lysis buffer. The samples were then centrifuged at 70,000× *g* at 4 °C for 1 h. The resulting green pellet at the bottom contained the thylakoid and was carefully removed and diluted with lysis buffer at 1:10 and centrifuged again at 110,000× *g* at 4 °C for 1 h. After removing the supernatant, the remaining pellet was diluted in 10 μL lysis buffer and stored at −80 °C.

### 4.4. Mass Spectrometer Analysis of Thylakoid Samples

The protein complement of the samples was isolated using SDS-PAGE by loading onto the resolving gel for only 1 cm. The protein complement was visualized by Coomassie staining and excised for proteomics analysis. The excised bands were destained, followed by disulfide reduction and carbamidomethylation of cysteine residues using iodoacetamide. Proteins were digested in gel using proteomics-grade trypsin and analyzed on a nano-reversed-phase liquid chromatography (LC) electrospray ionization tandem mass spectrometry (MS) system that consists of an UltiMate 3000 Nano LC System and an LTQ-Orbitrap Elite mass spectrometer (Thermo Fisher, San Jose, CA, USA). Chromatography was performed using a 20 cm × 75 μm ID column packed with XBridgeTM BEH C18 beads (2.5 μm and 130 Å). The resultant data were processed using Mascot software (Version 2.7.0) on our in-house Mascot server.

### 4.5. GLV Assays

GLV assays were performed as described in [9,26] with several modifications. For the determination of the effect of pH on the activity of the LOX10/HPL complex, we used potassium phosphate (KPi) buffers adjusted to the desired pH. After establishing an optimum at pH 5–6, all further experiments were performed at pH 6. Phenidone was pre-diluted in ethanol and added to the final concentration indicated in Figure 2. Ethanol was kept at the same level in all samples, including controls at 10%. Ca^2+^ and Mg^2+^ were used as the corresponding chloride salt and applied at a final concentration of 10 mM. EGTA and EDTA were also used at a final concentration of 10 mM. Both were pre-dissolved at pH 9 and then slowly titrated down to pH 6. Fatty acids (y-linolenic acid and stearic acid) were dissolved in ethanol and then added to a final concentration of 1 μg/μL with ethanol not exceeding 10%. Controls were assayed with similar ethanol concentrations.

### 4.6. GLV Analysis

For each experiment, similar segments from leaves at the same developmental stages were used. The segments were between 30 and 50 mg fresh weight and immediately placed in 2 mL screw-cap vials in liquid N_2_. After adding buffer (containing different effectors as described above) directly into the frozen vial and allowing it to freeze, we added about 600 mg of beads. By not being frozen in the buffer, the beads can move freely in the vials and disrupt tissues when placed in a PreCellys homogenizer set to 5600 shakes per minute for 25 s. The 2 mL microcentrifuge tubes were then unscrewed without removing the cap and immediately dropped into a 30 mL glass container while also releasing the cap into the glass container to avoid significant losses in volatiles. The glass containers were immediately capped. Volatile emissions were collected immediately from the tissue homogenate by inserting a volatile collection filter packed with 30 mg Hayesep Q absorbent (Supelco, Bellefonte, PA, USA) coupled to a vacuum at 0.3 L/min for 1 h as described previously [9,26]. Filters were then removed and eluted with 150 µL dichloromethane and 1000 ng of internal standard (nonyl acetate) was added. The analysis of damage-induced GLV production was performed on a Varian 3900 gas chromatograph coupled to a Varian Saturn 2200 mass spectrometer equipped with split–splitless capillary injector systems in electron impact mode (EI). The injection volume was 1 µL. The data collection, storage, and subsequent analysis were performed by using the Varian MS Workstation (Version 6.6). Helium at a constant flow rate of 1 mL/min was used as a carrier gas. The analyses of volatiles were performed on a fused silica capillary column (Equity™ 30 m × 0.25 mm inner diameter with a 0.25 µm-thick film of bonded methyl silicone). The GC was programmed as follows: 40 °C for 2 min, then at 15 °C/min to 250 °C. All of the injections were made in the split mode (1:20 split ratio). Compounds were identified by comparison to authentic standards (retention time and fragmentation). Due to the strong co-elution of Z3al and nHal, we used a selected ion (*m*/*z* 56) for stronger separation of the two compounds within a single peak area, as described in [26]. We then estimated the percentage of this ion in both compounds and used the calculated multiplication factor to determine the precise peak area. As a control, we also used the calculated area for nHal and subtracted it from the total peak area for both compounds.

### 4.7. Statistical Analysis

At least 4 biological replicates were performed per plant and per assay. Averages and standard deviation (SD) were calculated for each of the analyzed leaf segments. For pairwise comparisons, Student’s *t*-test was used (Microsoft Excel, Version 16.88), while for multiple comparisons, one-way ANOVA and Tukey’s test were applied (JMP statistical software, Version 17.2).

## Figures and Tables

**Table 1:** Non-photosynthetic proteins identified in thylakoid membranes of *Zea mays* chloroplasts.

Name	Protein View (NCBIprot Database 20181106)	emPAI	Identified Sequences (1-Letter Code)	M_r_
Lipoxygenase 2.3, chloroplastic [*Zea mays*] (LOX10)	PWZ26982.1	0.45	QLTFGATTLR FEVPEMIER SKLDPEVYGPAESAITK YTMEINALAR GEDGELELTIK SDEAVAADPELR DEPWWPVLDTR NMPVEEGGPGEEMEK	102005
Linolenate hydroperoxide lyase, chloroplastic [*Zea mays*] (HPL)	PWZ25671.1	0.4	TFAMDLLHR ASVGAMLDAVDAEFGKDDGSDK EGMPLVR DPEVFERPEEFVPER YDDFEVEGTSFTK	55519
Phospholipase A(1) DAD1, chloroplastic [*Zea mays*] (PLA1)	PWZ29609.1	0.09	AVSFGGPRVGNVAFR	43169

## Data Availability

Data are contained within the article.
